# Thiopurine Methyltransferase Activity and Thiopurine Metabolites in Inflammatory Bowel Disease

**DOI:** 10.1093/crocol/otaa062

**Published:** 2020-07-27

**Authors:** Wael El-Matary

**Affiliations:** Section of Pediatric Gastroenterology, Department of Pediatrics, Max Rady College of Medicine, Rady Faculty of Health Sciences and Children’s Hospital Research Institute of Manitoba, Winnipeg, Manitoba, Canada

Thiopurines (azathioprine, 6-mercaptopurine [MP], and 6-thioguanine [TG]) are purine analogs, used for their immunomodulator effect to maintain remission in persons with inflammatory bowel disease (IBD).^1–4^ They act through the incorporation of TG into DNA of T cells, resulting in DNA/RNA damage, apoptosis, and myelosuppression.^5^ Azathioprine is a pro-drug that undergoes enzymatic and nonenzymatic reduction in the liver in the presence of glutathione and releases MP and TG. 6-Mercaptopurine is converted to thioinosine monophosphate (tIMP) by hypoxanthine guanine phosphoribosyltransferase; tIMP is either converted to inactive inosine triphosphate (ITP by ITPase) or 6TImP (6 thioinosino-5′ monophosphate) by thiopurine methyltransferase (TPMT), which inhibits nucleic acid synthesis.^5, 6^ The enzyme, TPMT, provides a competitive pathway for inactivation of these drugs by thiol methylation.^6, 7^ A balance must be established between these 2 competing metabolic pathways in order to create enough TG. A serious adverse event of thiopurines is bone marrow suppression that could take place as a result of high levels of TG.^8^ Other adverse events include intolerance and hypersensitivity manifestations, such as vomiting, diarrhea, fever, myalgia, skin rashes, and hypotension.^7–9^ Rare idiosyncratic reactions include renal impairment, pneumonitis, and pancreatitis.^7–9^ Thiopurine-induced liver dysfunction secondary to methylated intermediate metabolites can present as elevated liver enzymes, cholestasis, hepatic veno-occlusive disease, and nodular regenerative hyperplasia.^9^

Increased cancer risk with the emergence of several concerning reports linking thiopurines to hemophagocytic syndrome and the fatal hepatosplenic T-cell lymphoma has certainly decreased utilization of thiopurines as a maintenance agent for persons with IBD, especially in the pediatric age group.^10, 11^

Thiopurine methyltransferase activity is inherited as a monogenic codominant trait.^6^ Distinct inheritable differences in levels of red blood cell TPMT have been detected among patients.^6, 8^ Patients with low or absent TPMT activity metabolize the drugs more slowly and have higher TG nucleotide concentrations, increasing the risk of developing severe, life-threatening bone marrow suppression if conventional doses are given.^7–9^ Measuring TPMT activity before prescribing immediate full doses of a thiopurine is a quality improvement indicator.^12, 13^ Approximately 0.3% of the population has undetectable TPMT activity, 11% low (intermediate) activity, and 89% normal activity. Between 30% and 60% of individuals with intermediate TPMT activity cannot tolerate a full thiopurine dose. They should be prescribed a 30%–70% reduction in thiopurine dose. Homozygous persons with undetectable TPMT activity require approximately 90% reduction in thiopurine dose or treatment with an alternative medication.^7–9^ However, measuring the TPMT activity at baseline is not a substitute for monitoring white blood cell counts throughout therapy as myelosuppression is still a threat in these patients.^8^

It is not clear if persons with normal to high TPMT levels need higher doses of thiopurines to establish a good therapeutic effect. Jonason et al^14^ examined the association of normal to high TPMT levels on TG metabolite concentrations in a cohort of 100 adult persons with IBD. The authors reported no effect on metabolite concentration with normal to high TPMT levels. Age, thiopurine dose, and body mass index predicted TG concentration. The authors identified TG cutoff of 224 pmol/8 × 10^8^ red blood cells as the threshold for maintaining remission, a finding that is concordant with what was previously published.^15^ Although the study is limited by the retrospective design and inability to measure adherence in many participants, the study highlighted an important finding as clinicians do not need to increase the dose of thiopurines in persons with IBD who have normal to high levels of TMPT.

## DATA AVAILABILITY

Data sharing is not applicable to this article as no new data were created or analyzed in this article.

## Notes

Funding: None.
